# Development of an Interdisciplinary Telehealth Care Model in a Pediatric Cystic Fibrosis Center

**DOI:** 10.1089/tmr.2021.0021

**Published:** 2021-10-12

**Authors:** Catherine Enochs, Amy G. Filbrun, Courtney Iwanicki, Haley Moraniec, Julie Lehrmann, Jourdan Stiffler, Sharyn Dagher, Chris Tapley, Hanna Phan, Rebekah Raines, Samya Z. Nasr

**Affiliations:** ^1^ Department of Pediatrics, Michigan Medicine, C.S. Mott Children's Hospital, Ann Arbor, Michigan, USA.; ^2^ Department of Clinical Pharmacy, College of Pharmacy, University of Michigan, Ann Arbor, Michigan, USA.

**Keywords:** telehealth, telemedicine, cystic fibrosis, interprofessional, interdisciplinary

## Abstract

**Background:** People with cystic fibrosis (PCF) have unique physical and emotional needs, which are best met through interdisciplinary care (IDC). In the midst of the pandemic, our center aimed to begin a telehealth care model with an objective to increase successful care visits from baseline of 0–95% by June 26, 2020, including meeting cystic fibrosis (CF) care standards of IDC visits that are coproduced through agenda setting with PCF.

**Methods:** Shifting IDC for pediatric CF patients to telehealth was part of a quality improvement initiative. Our team used asynchronous virtual visits (VVs), with the IDC team members' VVs done on different days than the physician's. Multiple plan–do–study–act cycles were completed to address evolving telehealth needs, including IDC team member flow logistics, communication with PCF, and surveying PCF for the patient perspective. Rates of IDC and agenda setting were measured from March 16, 2020 to June 26, 2020.

**Results:** IDC VVs were at 86% in March 2020 with fluctuations until mid-May when we reached 100% and achieved sustainability. Agenda setting was reached at 100% and maintained. With continued effort, an additional 46.3% of PCF registered for the patient portal, totaling 90.6% with access. Our survey revealed 100% of PCF were able to see IDC team members that they needed to, with 87% “extremely satisfied” and 13% “somewhat satisfied” with their telehealth experience.

**Conclusions:** Successful telehealth in pediatric CF IDC can be achieved through continuous communication, optimal utilization of available technologies, and may help foster unique opportunities to help improve health outcomes.

## Introduction

Cystic fibrosis (CF) is an inherited autosomal recessive genetic disorder that causes dysfunction in the Cystic Fibrosis Transmembrane Conductance Regulator protein. It affects many organ systems, but primarily affects the lungs and gastrointestinal tract. Various treatments are needed for the care of people with cystic fibrosis (PCF), including chronic management as well as treatment of acute pulmonary exacerbations.^1–4^ Children with CF meet the widely recognized definition of children and youth with special health care needs, given their unique physical, developmental, behavioral, or emotional needs.^5^ Owing to the complexity of the disease, care of PCF requires an interdisciplinary care (IDC) team of CF specialists, including pulmonologists (MD), registered nurses (RN), social workers (SW), clinical pharmacists (PharmD), registered dietitian nutritionists (RDN), respiratory therapists, physical therapists (PT), and psychologists. In addition, a care model that incorporates IDC improves patient outcomes, as we have documented in our center.^6^ Traditionally, such IDC is provided through in-person clinic visits with direct patient care responsibilities from many or all noted disciplines. In this study, we describe a successful quality improvement (QI) initiative in translating our patient-focused IDC model into telehealth at the Michigan Medicine Pediatric Cystic Fibrosis Center. The center provides preventative and acute care to nearly 275 pediatric patients, ages 0–21 years, from Michigan, surrounding states, and internationally.

**FIG. 1.**
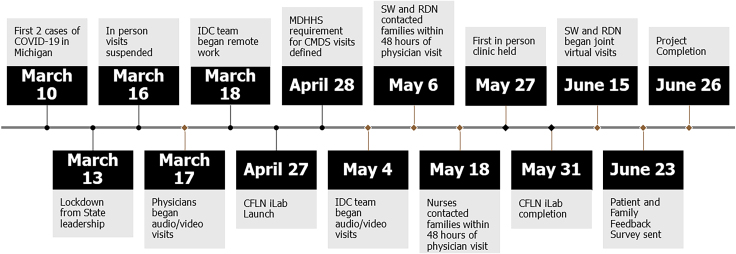
2020 events timeline. CFLN, Cystic Fibrosis Learning Network; CMDS, Children's Multidisciplinary Specialty; COVID-19, coronavirus disease 2019; IDC, interdisciplinary care; iLab, innovation laboratory; MDHHS, Michigan Department of Health and Human Services; RDN, registered dietitian nutritionists; SW, social workers.

The coronavirus disease 2019 (COVID-19) began with documented cases in Wuhan, China in December 2019 (Fig. 1).^7^ On March 10, 2020, Michigan saw its first two cases of presumptive-positive COVID-19 cases.^8^ Beginning March 16, 2020, in-person visits for PCF were suspended at the CF center, with our institution mandating that virtual visits (VVs), either by audio only through phone or by audio/video, be used as the primary visit modality for all clinics. MD audio/video visit training was implemented rapidly, resulting in audio/video visits beginning March 17, 2020. The IDC team continued virtual care through phone contact until training was completed for audio/video visits by May 4, 2020. During this time, the pediatric CF program was also beginning work as part of the Cystic Fibrosis Learning Network (CFLN), a multicenter QI network of accredited CF Centers funded by the CF Foundation. Launch of the telehealth innovation laboratory (iLab), a multicenter QI collaborative through CFLN to implement multidisciplinary telehealth care, began April 27, 2020.

The specific workflow at a given center is a primary consideration in transitioning CF IDC into telehealth. For example, if a center has dedicated CF clinic days with one physician, synchronized visits where the various disciplines see patients in a given sequence may be feasible. In contrast, when multiple physicians are conducting clinics at the same time, synchronized visits with other team members are more challenging, and asynchronous IDC may be more fitting. The latter is the case in our center, and we found asynchronous IDC to be most appropriate, with non-MD disciplines completing virtual patient visits in the days surrounding the MD visit. Although feasibility of IDC in telehealth has been documented both in CF and non-CF populations, the clinic workflows, results, and outcomes have been varied.^9–12^ Successful synchronous telehealth implementation by other CFLN CF centers has also been documented; however, literature regarding asynchronous telehealth implementation in the care of PCF is currently lacking.^13,14^

We describe our care model of transitioning to telehealth urgently to respond to patients' care needs during a pandemic. The QI project was done in collaboration with the CFLN. The aim was to deliver IDC with previsit agenda setting through telehealth, while complying with CF Foundation, state health official and institutional guidelines. The objective was to increase successful care visits from baseline of 0% to 95% by June 26, 2020. Successful visits would include documented contact by at least one of the IDC team members and a visit agenda coproduced with patients and families.

## Materials and Methods

We reviewed all scheduled patients cared for by our center between March 16 and June 26, 2020. This project met the definition of QI at our institution, and as such was determined to be not regulated by our Institutional Review Board.

### IDC logistics and coordination

To facilitate IDC coordination and help review the needs of PCF before their appointments, the team had been conducting weekly pre-clinic rounding meetings (CF huddle) since 2017. During these meetings that included all non-MD IDC disciplines, patients scheduled for clinic visits through the following week were reviewed. Pertinent information from huddle was electronically communicated securely to the IDC team as well as posted in the provider clinic workspace and referenced by all staff during clinic. Our center's weekly CF huddles continued using a secure virtual platform (Zoom Video Communications Inc., San Jose, CA) and had no lapse in occurrence during the shift into telehealth. Communication of the pertinent information noted in huddle was shifted to entirely electronic, since no central clinic space was being utilized for conducting telehealth visits.

### Collaboration with patients and caregivers

One of the tools that IDC team members utilized to communicate with PCF was the patient portal within our electronic medical record (EMR), EPIC Systems Corporation (Verona, WI). To increase use of a Health Insurance Portability and Accountability Act (HIPAA) compliant virtual platform, our clinic began helping patients sign up for the patient portal to gain access to secure audio/video visit software through Zoom. Our goal was to have all patients signed up for the patient portal by the end of our project. Administrative assistants instructed patients on portal registration and IDC team members provided technology support and encouraged patients to register.

To improve communication and keep our patients informed, we created a report that allowed us to rapidly send communications to our families through the patient portal. As we implemented changes to the clinic and held a town hall event for transparency, the patient portal communications were a way to keep PCF informed of changes and recommendations and to provide supportive resources.

Seeking to capture measurable data on the patient and family experience with telehealth, our core QI team adapted, with permission, a clinical tool from the University of Alabama. The patient and family feedback survey (PFFS), an anonymous 21-question survey was created using a web-based survey tool (Qualtrics^®^, Provo, UT) and sent either through e-mail or patient portal to PCF. For those who had more than one child with CF, we asked them to complete a survey for each child. The PFFS was sent out initially to all patients, then it was sent 1–4 weeks following each MD appointment thereafter. The survey asked about IDC provided, telehealth experience, and satisfaction, and if all questions or concerns were addressed, among other questions. It did not differentiate patient versus parent responses or indicate repeat surveying for tracking of changed responses over time.

**FIG. 2.**
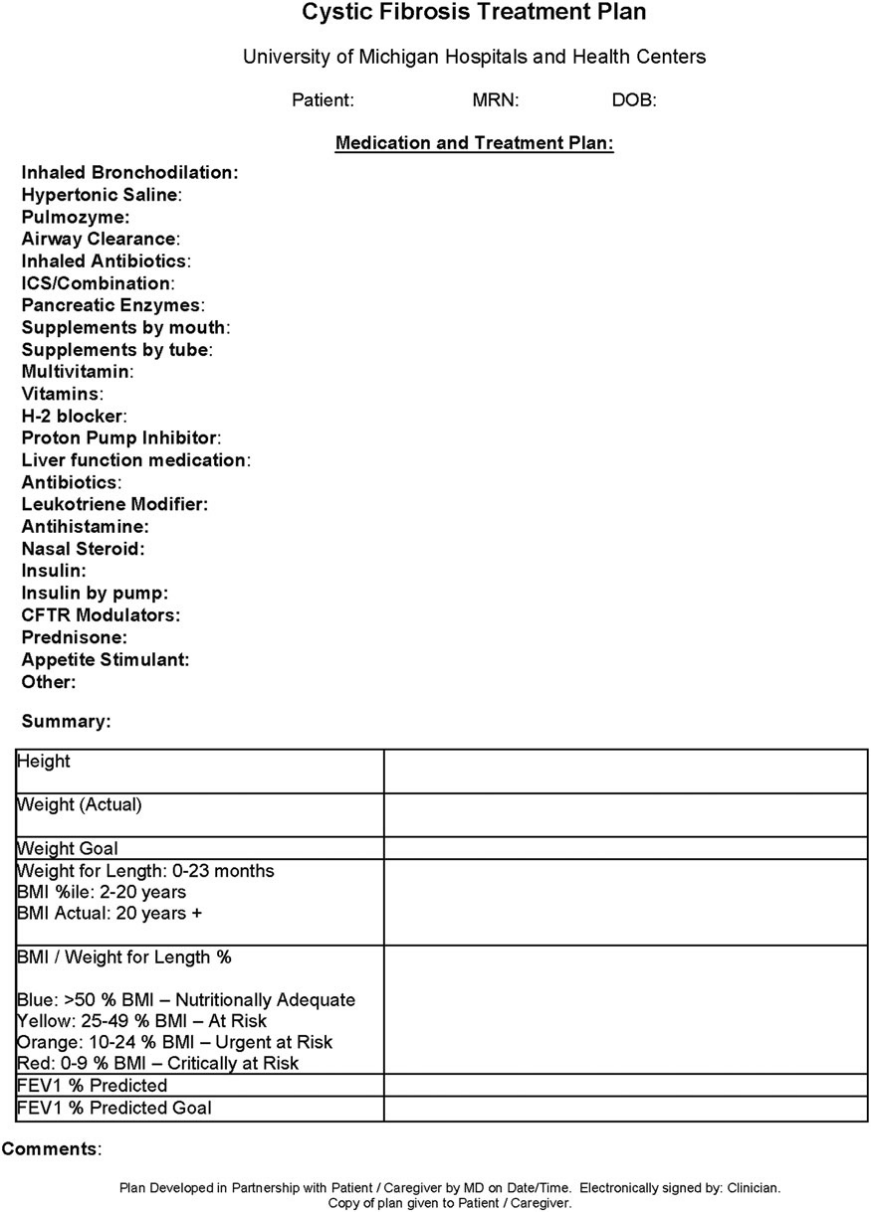
Cystic fibrosis treatment plan. BMI, body mass index; DOB, date of birth; FEV1, forced expiratory volume in 1 second; MD, pulmonologists; MRN, medical record number.

Another communication tool, used after the visits, was our CF treatment plans, which have been used for many years. These plans include medication lists and changes, statistics and goals from the visit and recommendations made during the visit (Fig. 2). Since these were already created within the EMR, switching to patient portal delivery did not require excessive logistical planning given our ability to communicate through patient portal. Since RDN was seeing PCF in the days leading up to the MD visit, they began updating the CF treatment plan in their EMR encounter. As this documentation saved in the EMR, it was readily available for the RN or PharmD to complete and send to the patient after the MD visit.

### QI and data processes

As a QI initiative, we utilized widely recognized QI tools such as plan–do–study–act (PDSA) cycles, process flow diagrams, patient satisfaction surveys, and data collection and review.^15^ The core QI team consisted of a Physician Lead, two Quality Improvement Co-Leads, a parent of a child with CF (patient/family partner [PFP]), and representatives from all CF IDC disciplines. Implementation of each PDSA cycle was discussed with all QI team members including the PFP. Weekly QI meetings were held while PDSA cycles were developed and implemented, and adaptation or adoption was considered. Weekly data were submitted to CFLN for tracking of agenda setting and IDC visits. Logistical support for data collection, tracking, and reporting were provided by the CFLN. To keep track of the members of the IDC that met with PCF, SW developed a secure spreadsheet for the team, available for editing through our institution contracted HIPAA-compliant online document sharing, cloud storage platform (MBox, Box, Inc., Redwood City, CA). Team members documented both attempted and successful contact with PCF. This method also helped to better track patient encounters for billing purposes and IDC data tracking.

**FIG. 3.**
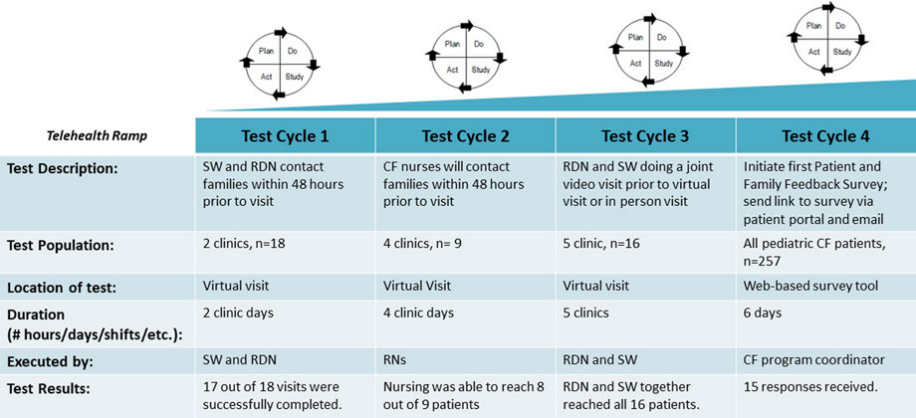
Telehealth PDSA ramp summary. CF, cystic fibrosis; PDSA, plan–do–study–act; RN, registered nurses.

IDC was defined as having audio/video or audio contact with one or more providers outside of the MD or RN surrounding the MD visit. Patients and families who declined seeing a team member or failed to return contact attempts were still counted as having had the opportunity for IDC in the data submitted to CFLN but are not included in successful VVs in our results. Agenda setting was defined as verbal or written communication of the patient and family's goals, concerns, or topics to discuss for their visit. PDSA tracking was used and communicated to the core QI team and the CFLN regularly (Fig. 3).

## Results

At the onset of the pandemic in March 2020, all CF visits were transitioned to VVs (Fig. 1). Physicians were the first to complete virtual care training per institutional guidance, followed by other disciplines. Many of our usual clinic processes such as mental health screening and Michigan Department of Health and Human Services (MDHHS) Children's Multidisciplinary Specialty (CMDS) clinic billing were initially placed on hold as we adapted to a new telehealth clinic flow. On April 28, 2020, the MDHHS CMDS clinic billing requirements were updated, which included IDC team members speaking with patients either through phone, audio/video, or in-person within 48 h of a physician's visit. This prompted our first two PDSA cycles, which showed that RDN, SW, and RN were able to connect with almost all patients within the 48-hr time frame by phone or audio/video visit (Fig. 3). In response to advice from our patient and family advisory council (PFAC), RDN and SW began PDSA 3, which included combining their visits, when able, to decrease the number of independent visits. They began with combining phone visits until audio/video visit training was completed. This improved scheduling and decreased the burden of attending multiple visits for PCF and their families. With 100% of attempts being successful, this was also adopted to our clinic flow. At the time, RN and PharmD did not have audio/video visit capabilities or training and thus were contacting patients over the phone in the days before an MD visit. PT also transitioned to a full telehealth model and was available on a consultative basis.

PDSA 4 was initiated to elicit feedback on our IDC clinic process from PCF and their families. Of the PFFS responses received in the first week (*N* = 15), all responded that they were able to see the members of the IDC team that they needed to see, with 87% “extremely satisfied” and 13% “somewhat satisfied” with their telehealth experience. When asked if all questions and concerns were addressed in the telehealth visit, 100% responded “Yes.” We have continued weekly surveying of PCF seen in clinic beyond the project period. Surveys from July 2020 to June 2021 have revealed similar results (*N* = 64), with 97% reporting they were able to see all needed IDC team members. Most respondents also felt satisfied utilizing telehealth services, with 81% reporting “extremely satisfied” and 8% reporting “somewhat satisfied.” Similar to the initial PFFS responses, 98.33% of PCF stated “Yes” to having all their questions or concerns addressed in their telehealth visit, although four respondents did not complete this question.

**FIG. 4.**
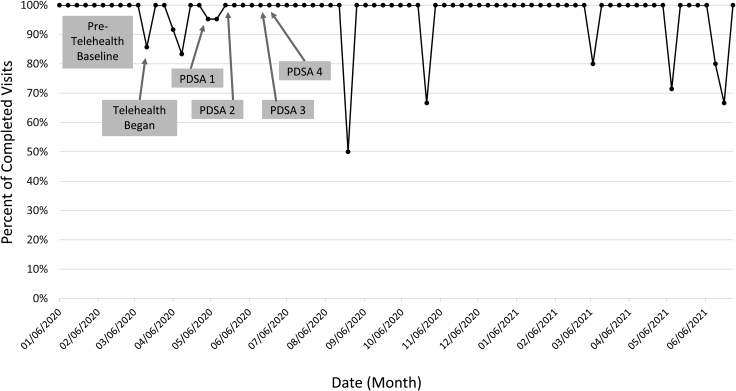
Virtual visits with interdisciplinary care.

In the 10 weeks before our project, from January 6 to March 13, 2021, we had a total of 232 in-person visits with a mean of 23 (range 16–31) visits per week and having a success rate of 100% (Fig. 4). VVs that included IDC were 86% in mid-March 2020 with fluctuations in percentages through mid-May 2020, when 100% IDC visits was achieved, and sustainability reached and maintained through June 26, 2020. During the 15-week project, overall, we had 187 fully telehealth visits with a mean of 12 (range 1–30) visits per week. Of those, 179 had IDC, resulting in a 95% success rate. In the 51 weeks following the end of the project our center had 194 telehealth visits with a mean of 4 (range 0–9) visits per week. With 187 visits having IDC, we had an overall success rate of 96%. Agenda setting goal of 95% of visits being coproduced was reached with all our patients, as all were able to have the opportunity and ability to discuss their goals for the visit either verbally or through the patient portal before their visits.

Another goal was to have all PCF register to the patient portal. At the start of the project 43.9% of PCF had activated their patient portal. During the project period, an additional 35.7% of patients registered for the portal, for a total of 79.6% of our population having access by July 2020. Between July and December 2020, another 11% activated their portals for a total of 90.6% of PCF seen at our center having access to the patient portal.

## Discussion

The sudden onset of restrictions in Michigan to combat the global COVID-19 pandemic required our clinic to make drastic changes to patient care delivery in a short period of time. Since our clinic had not previously been utilizing telehealth, it took a targeted, engaged, and dedicated group of IDC team members to pivot our care and develop, implement, evaluate, and adjust the new telehealth model. PCF seen at our center showed flexibility and understanding as they adapted to changes to the care model. Our PFP provided the important patient perspective throughout the project, engaging with the team candidly.

Input from PCF is a valuable evaluation tool. Our survey to patients initially provided a response rate of 5%, which is lower than some studies and a limiting factor.^16,17^ Anecdotal feedback from our PFP and CF PFAC provided potential reasoning for low response rate, referencing survey, and patient portal message fatigue. The survey beyond the project time frame included both hybrid and fully telehealth visits while also providing anonymity for respondents. The hybrid visits provided virtual care by RDN, SW, and eventually PharmD, whereas the remaining care was done face-to-face. We were unable to distinguish responses regarding fully telehealth visits versus hybrid, which was another limitation of the survey. Therefore, we have reported the cumulative responses for both visit types. In a multicenter study of 11 CF Centers (4 adult and 7 pediatric), in 2021 Jaclyn et al.^18^ reported similar results to what our survey showed. Of the pediatric programs, 99% of patients/parents felt all issues and concerns were addressed, 93% were able to see desired disciplines, and 72% had a high level of satisfaction.^18^

Owing to the return of PCF to the clinic through hybrid visits, the number of fully telehealth visits dropped drastically. With a low number of fully telehealth visits per week in the year following the project, PCF who did not receive IDC had a greater impact on the weekly results. In the year following the project, all PCF who did not receive IDC had attempted contact by multiple IDC team members and either did not respond to attempts or did not complete scheduled visits with the IDC team.

Although the virtual model was created out of necessity, it will continue moving forward in some capacity. The IDC team felt that it improved patient care, especially for some of the team members (SW, RDN, and PharmD) since they can do more with their visits and offer more frequent follow-up. VVs with these disciplines before MD visits have provided additional opportunities for engagement with PCF in care, education, and follow-up without worrying about the rush of the clinic visits. These added opportunities have proven to be extremely beneficial, as many patients and families have needed additional support due to the numerous stressors the pandemic has placed on them. This is consistent with national survey results that Perkins et al. published in 2021, which stated IDC team members felt efficiency, clinician–patient relationship and visit satisfaction were all improved with telehealth use.^19^

Building redundancy into our system with multiple opportunities for agenda setting before the MD visit was critical to achieving our goal of 100% agenda setting. Each IDC team member used agenda setting language with PCF and followed up with communication to the team. PFP feedback supported this method, reflecting that each provider addressing the needs for the visit was appreciated and did not feel redundant, but instead felt like the team communicated well.

Telehealth offers the unique opportunity to care for patients without adding the burden of travel or more time off work or school. In specialty care, some of our patients travel long distances to attend clinics, so overnight hotel accommodations must be arranged by the family. Telehealth has also offered a more open conversation with some PCF, especially as children are more comfortable in their homes. Some children who have historically been more withdrawn and disengaged in face-to-face clinics are more engaged with the IDC team during VVs.

However, telehealth should not replace all in-person care. The use of telehealth posed some difficulties in assessment capabilities that limit its use as the sole mode of providing CF care. Ongoing identification of respiratory pathogens is usually done by throat/sputum culture in clinic on a quarterly basis. Respiratory cultures were suspended for VVs. Similarly, routine annual CF laboratory work and chest radiographs were suspended until in-person clinics resumed. The inability to measure lung function through spirometry posed a significant challenge, as well. Our center suspended all outpatient spirometry testing until face-to-face visit restrictions were lifted on May 27, 2020. In the same time frame, the CF Foundation began providing portable home spirometry devices to PCF through ZEPHYRx^®^ (Troy, NY), for remote patient monitoring.

A potential improvement to the CF care model would be to adopt a hybrid model, utilizing both telehealth and in-person visits throughout a given year. This will allow flexibility for patients and families while still allowing for close monitoring of PCF and adhering to CF care guidelines, including chest radiographs, measurement of lung function, and collection of sputum or throat cultures as well as performance of a physical examination. Monitoring outcome measures could reveal if telehealth impacts outcomes over time.

Some of the current literature regarding implementation of telehealth in CF centers has focused their model on synchronous visits.^13,14^ Literature is lacking on the use of asynchronous visits to successfully provide IDC to PCF, despite the slight preference for these visits among CF center clinicians.^19^ With varying clinic logistics across CF centers, asynchronous visits have provided different opportunities for care to the IDC team and PCF. Appointment length is not limited by time in the same way it is during a face-to-face clinic visit, providing more opportunities for in-depth assessments and interventions with the IDC team, as well as increased follow-up frequency. PCF and their families have provided positive feedback about this model as well, with comments on our PFFS indicating improvement in the patient experience.

Most of our patients are active on the patient portal, which provides our virtual platform for visits; however, 9.4% of our patients do not have access. These patients have been reachable by phone or nonportal audio/video, making VV still possible without portal access. Socioeconomic factors, access to reliable internet, and/or devices with cameras may have been factors in lack of access for VV using the patient portal. Patients also require multiple appointments in 1 week, which can be difficult depending on a family's support system and employment situation. Depending on a clinic's patient population, clinic location, and patient socioeconomic factors, asynchronous VV may provide more barriers to care for some PCF.

This project did encounter some limitations. The number of patients participating in the project was relatively small. Although the CFLN iLab provided guidance and support for multiple centers regarding agenda setting and IDC with telehealth implementation, each center developed and adjusted their model based on their clinic. This provided advantages to modify more easily but decreased the number of patients and thus the strength of the project. PCF also had varying levels of comfort and time limitations regarding telehealth tools and time commitment. Another limitation is the low PFFS responses. In addition, survey links sent in the patient portal cannot provide hyperlinks, so PCF had to highlight, copy, and paste the link address into a browser to complete it. Although our staff were successful in coproducing a clinic agenda verbally with PCF at the beginning of a clinic visit, institutional technology support and delays limited our ability to do agenda setting through the patient portal regularly during the project period. Starting mid-2021, we now have a previsit questionnaire automatically populate in the visit electronic check-in process for PCF before all VVs, which assesses goals and concerns, documenting responses in the EMR for access by the IDC team.

As clinic restrictions were lifted, clinic visits remained asynchronous as our care reached sustainability, but new hybrid visits were developed for face-to-face visits. Utilizing both fully virtual and hybrid asynchronous visits would be the new normal for our clinic through 2020 and into 2021. The schedule flexibility of PCF regarding asynchronous appointment availability in the future is unknown, as many schools and employers evaluate the role of remote work moving forward. While COVID-19 vaccine distribution continues and as restrictions in states begin lifting, PCF and their families may return to in-person work or school.

## Conclusions

Our pediatric IDC model changed drastically over the course of the COVID-19 pandemic. Although this transition has presented a number of challenges, with the application of numerous PDSA cycles, we now have a consistent clinical workflow that allows us to coordinate non-MD discipline IDC visits with the MD's clinic visits using asynchronous telehealth visits. Although patient satisfaction, IDC, and agenda setting have been shown to be sustainable with telehealth, additional data are needed to evaluate potential impact on patient outcomes. Study of measures such as weight, body mass index, adherence, and lung function would demonstrate whether regular care through telehealth is in fact a sustainable health care model for routine use outside of a global pandemic. The successful implementation of asynchronous IDC telehealth in this pediatric CF care center is a model that other CF centers, primary care or specialty care providers may consider for care from routine to sick visits, patient/caregiver education, or follow-up post-intervention.

## Abbreviations Used

BMI: body mass index
CF: cystic fibrosis
CFLN: Cystic Fibrosis Learning Network
CMDS: Children's Multidisciplinary Specialty
COVID-19: coronavirus disease 2019
DOB: date of birth
EMR: electronic medical record
FEV1: forced expiratory volume in 1 second
HIPAA: Health Insurance Portability and Accountability Act
IDC: interdisciplinary care
iLab: innovation laboratory
MD: pulmonologists
MDHHS: Michigan Department of Health and Human Services
MRN: medical record number
PCF: people with cystic fibrosis
PDSA: plan–do–study–act
PFAC: patient and family advisory council
PFFS: patient and family feedback survey
PFP: patient/family partner
PharmD: clinical pharmacist
PT: physical therapist
QI: quality improvement
RDN: registered dietitian nutritionist
RN: registered nurse
SW: social worker
VV: virtual visit
